# Right heart catheterization using metallic guidewires and low SAR cardiovascular magnetic resonance fluoroscopy at 1.5 Tesla: first in human experience

**DOI:** 10.1186/s12968-018-0458-7

**Published:** 2018-06-21

**Authors:** Adrienne E. Campbell-Washburn, Toby Rogers, Annette M. Stine, Jaffar M. Khan, Rajiv Ramasawmy, William H. Schenke, Delaney R. McGuirt, Jonathan R. Mazal, Laurie P. Grant, Elena K. Grant, Daniel A. Herzka, Robert J. Lederman

**Affiliations:** Cardiovascular Branch, Division of Intramural Research, National Heart, Lung, and Blood Institute, National Institutes of Health, Building 10, Room 2C713, Bethesda, MD 20892-1538 USA

**Keywords:** Interventional MRI catheterization, Right heart catheterization, Guidewire, Cardiac catheters, Medical device heating, Real-time MRI, Invasive hemodynamics, Spiral MRI, Cardiovascular magnetic resonance

## Abstract

**Background:**

Cardiovascular magnetic resonance (CMR) fluoroscopy allows for simultaneous measurement of cardiac function, flow and chamber pressure during diagnostic heart catheterization. To date, commercial metallic guidewires were considered contraindicated during CMR fluoroscopy due to concerns over radiofrequency (RF)-induced heating. The inability to use metallic guidewires hampers catheter navigation in patients with challenging anatomy. Here we use low specific absorption rate (SAR) imaging from gradient echo spiral acquisitions and a commercial nitinol guidewire for CMR fluoroscopy right heart catheterization in patients.

**Methods:**

The low-SAR imaging protocol used a reduced flip angle gradient echo acquisition (10° vs 45°) and a longer repetition time (TR) spiral readout (10 ms vs 2.98 ms). Temperature was measured in vitro in the ASTM 2182 gel phantom and post-mortem animal experiments to ensure freedom from heating with the selected guidewire (150 cm × 0.035″ angled-tip nitinol Terumo *Glidewire*). Seven patients underwent CMR fluoroscopy catheterization. Time to enter each chamber (superior vena cava, main pulmonary artery, and each branch pulmonary artery) was recorded and device visibility and confidence in catheter and guidewire position were scored on a Likert-type scale.

**Results:**

Negligible heating (< 0.07°C) was observed under all in vitro conditions using this guidewire and imaging approach. In patients, chamber entry was successful in 100% of attempts with a guidewire compared to 94% without a guidewire, with failures to reach the branch pulmonary arteries. Time-to-enter each chamber was similar (p=NS) for  the two approaches. The guidewire imparted useful catheter shaft conspicuity and enabled interactive modification of catheter shaft stiffness, however, the guidewire tip visibility was poor.

**Conclusions:**

Under specific conditions, trained operators can apply low-SAR imaging and using a specific fully-insulated metallic nitinol guidewire (150 cm × 0.035” Terumo *Glidewire*) to augment clinical CMR fluoroscopy right heart catheterization.

**Trial registration:**

Clinicaltrials.gov NCT03152773, registered May 15, 2017.

**Electronic supplementary material:**

The online version of this article (10.1186/s12968-018-0458-7) contains supplementary material, which is available to authorized users.

## Background

Cardiovascular magnetic resonance (CMR) fluoroscopy catheterization allows simultaneous measurement of chamber pressure and cardiac output, alongside characterization of cardiac tissue, cardiac anatomy and cardiac function in a single procedure [1].

To date, clinical CMR fluoroscopy heart catheterization has relied on polymer catheters that are visualized solely by the contents of distal balloons filled with gas or contrast agents [2–6]. As a result, only catheter tips and not shafts are visible during CMR fluoroscopy, which hampers catheter navigation, especially in patients with challenging anatomy and physiology, such as surgically corrected or uncorrected congenital heart disease, enlarged right sided heart structures and severe valvular regurgitation. Until now, metallic guidewires, an important adjunct for X-ray fluoroscopy catheterization, have not been used during CMR fluoroscopy catheterization out of concern that long conductive structures will heat by coupling electrically with radiofrequency (RF) excitation pulses [7, 8]. Glass and other non-metallic guidewires have been reported for CMR catheterization but suffer insurmountable fragility that will likely preclude wide adoption [9–12]. Non-conductive metallic guidewires have been reported that resist heating during CMR but that are not yet commercially available [13].

We hypothesized that standard commercial metallic guidewires could be safely used in patients by reducing CMR excitation energy (also known as low specific absorption rate (SAR) imaging) [14]. We defined conditions of use for a specific commercial nitinol guidewire having desirable insulation properties, used in combination with reduced excitation energy and prolonged readout times to reduce RF-induced heating. These consisted of reduced flip angle α (to 10^o^ during gradient echo from 45^o^ during balanced steady-state free precession (bSSFP), a long repetition time (TR) spiral readout instead of a short TR rectilinear readout (TR 10 ms vs 2.98 ms), and a specific 150 cm nitinol guidewire having fully-insulated proximal and distal tips (*Glidewire* 0.035″, Terumo). Using these parameters, we tested guidewire heating under exaggerated geometry conditions in vitro, in situ in post-mortem swine, and finally used this guidewire in consenting research subjects undergoing clinical CMR fluoroscopy catheterization.

## Methods

### CMR

Procedures were performed in a combined X-ray and CMR fluoroscopy suite (Artis Zee and Aera 1.5 T, Siemens Healthineers, Erlangen, Germany) for both animals and patients. The CMR fluoroscopy room incorporates LCD projectors, wireless sound-suppression communication headsets (*IMROC IR*, Optoacoustics, Or Yehuda, Israel), and an artifact-suppressing patient physiology interface (*PRiME*, *nhlbi-mr.github.io/PRiME/*) [15] for a standard high-fidelity hemodynamic recording system (*Sensis*, Siemens Healhtineers).

CMR fluoroscopy used one of two possible pulse sequences: **low-SAR with metallic guidewire** (spiral gradient echo (GRE), 16 spiral arms per frame, matrix 128 × 128, TR 10 ms, TE 0.86 ms, flip angle 10^o^, FOV 400 mm, slice thickness 8 mm, spatial resolution 3.125 mm × 3.125 mm, temporal resolution 160 ms per frame); or **normal-SAR without metallic guidewire** (rectilinear bSSFP, matrix 120 × 160, TR 2.98 ms, TE 1.49 ms, flip angle 45^o^, field-of-view (FOV) 400 mm, slice thickness 8 mm, GRAPPA acceleration rate 2–4, spatial resolution 3.33 mm × 2.5 mm, temporal resolution 89.4 ms - 178.8 ms per frame). Guidewire heating is proportional to the square of the flip angle and inversely proportional to TR [14] and therefore the parameters used for low-SAR protocol theoretically generated a 67 fold reduction in heating compared to the normal-SAR protocol. A saturation pre-pulse was used to improve visibility of gadolinium filled balloons interactively. The low-SAR sequences used a partial saturation pulse with flip angle 45° [16] and the normal-SAR sequence used a dark blood saturation preparation. Imaging was performed in normal SAR operating mode, limiting time-averaged whole body SAR to 2 W/kg.

### Catheter and guidewire devices

We selected a polymer balloon-wedge endhole catheter with a rigid distal curve that in our experience is useful to navigate cardiac chambers (Swan-Ganz Flow-Directed Monitoring Catheter, T-tip, Dual-Lumen including inflation port, Model T111F7, 7Fr × 110 cm, 0.038″ lumen, Edwards Lifesciences, Irvine, California, USA) [2, 3], but had other shapes available on standby (Balloon Wedge Pressure Catheter, Model AI-07127, Arrow-Teleflex, Reading, Pennsylvania, USA). Balloon catheters were filled with gadobutrol (diluted 0.5% from stock) or air.

We selected a specific guidewire for its materials and insulation properties. The guidewire (*Glidewire* Model GR3506, Terumo Corporation, Tokyo, Japan, 0.035″ × 150 cm, standard stiffness, 3 cm flexible tip, angled tip configuration) has a single nitinol core and is completely insulated with expanded polytetrafluoroethylene (ePTFE) without exposed gaps. By contrast, a popular comparator PTFE-coated nitinol guidewire (*Nitrex*, Medtronic,Minneapolis, Minnesota, USA, Covidien EV3, for example 0.035″ × 145 cm Model N351452, Medtronic) has exposed and un-insulated tips, which we consider unsafe for use in CMR.

### In vitro experiments

Confidence in freedom from heating was established with in vitro experiments using an ASTM 2182 gel phantom. This gel phantom exaggerates heating and *in vivo* we expect less heating because of blood flow cooling, the central position of the vessels and convective heat dissipation.

Guidewire temperature was evaluated for a range of guidewire configurations, as summarized in Table 1; catheter position relative to guidewire, guidewire looping, guidewire length and guidewire position within the bore were among conditions explored. A fiber optic temperature probes and signal conditioner with ±0.3 °C accuracy were used for temperature measurements (OpSens Solutions Inc., Quebec, Canada). The temperature probe was affixed to the guidewire using a polyimide channel fastened with heat shrink tubing [17], or positioned using a homebuilt modular positioning system that supports both the guidewire and temperature probe in contact.

**Table 1:** Guidewire configurations tested during in vitro experiments. Experiments were performed in the ASTM-2182 phantom using Terumo *Glidewires* and Medtronic *Nitrex* in a 70 cm bore 1.5 T MRI system (Aera, Siemens, Erlangen, Germany)

Property	Experimental details	Maximum Heating Condition
Catheter insulation	• Heating at guidewire tip and catheter tip were measured simultaneously • Guidewire position relative to catheter: 10 cm, 5 cm, 2 cm, 1 cm (guidewire extended), 0 cm (tips aligned), − 2 cm, − 5 cm (guidewire retracted inside catheter).	• 1 cm guidewire extension; the change in insulation is similar to that of an exposed metallic tip.
Guidewire Length	• Terumo *Glidewires* of lengths 150 cm, 180 cm and 260 cm with catheter insulation (1 cm exposed guidewire)	• 260 cm guidewire for Terumo *Glidewires*
Guidewire position	• The temperature distribution of this phantom with this CMR system has been mapped previously [17]	• Positioned near the edge of the bore (> 12 cm off-isocenter) and near the surface of the gel (> 5 cm depth).
Insertion length	• Insertion lengths 15-65 cm into the ASTM gel phantom were tested for the three lengths of Terumo *Glidewire* with catheter insulation (1 cm exposed guidewire)	• 45 cm–55 cm insertion length for all guidewire lengths.
Looping	• Loops were created in segments of bare wire, as is common in the body, in the coronal plane • Loop diameter = 5 cm and single, double, triple and quadruple loops were compared. • Temperature was measured both at the tip of the guidewire and the contact point of the loops, where a second hotspot is formed.	• Temperature was always higher at the guidewire tip compared to the loop contact point and temperature in both locations increased with increasing number of loops. • Using the bare wire with no catheter insulation, generates lower temperatures and looping was not investigated further

### Animal experiments

Animal experiments were approved by the Institutional Animal Care and Use Committee. Guidewire heating was assessed post-mortem in a 47 kg swine to replicate right-heart catheterization guidewire geometry without blood flow cooling. Temperature was measured at the tip of the selected *Glidewire* extended 1 cm from a 7F catheter (Goodale-Lubin, Medtronic).

### Patient catheterization

The clinical research protocol was approved by the Institutional Review Board (NCT03152773). CMR fluoroscopy heart catheterization is a standard medical procedure at our institution. Consecutive patients undergoing medically necessary right heart catheterization between August and November 2017 were invited to participate. All gave written informed consent. Candidates were excluded for cardiovascular instability, pregnancy or nursing, or ineligibility for CMR. Those with an estimated glomerular filtration rate < 30 mL/min/1.73m^2^ were enrolled, but balloon catheters were filled with air rather than dilute gadolinium contrast.

Under moderate sedation, subjects underwent measurement of left ventricular pressure under X-ray guidance, followed by the research CMR fluoroscopy catheterization. Interventional procedures were performed before or after the research study, such as radial artery access or coronary intervention, and heparin anticoagulation was administered at the discretion of the physician. Baseline CMR was performed to assess anatomy, chamber dimensions and biventricular function.

For guidewire CMR fluoroscopy catheterization, time-to-enter each of four targeted chambers (superior vena cava, main pulmonary artery, right and left pulmonary arteries) was recorded. At their discretion, operators interactively retained or extended the guidewire beyond the catheter tip, to probe and enter the target chamber.

The guidewire was introduced following catheter positioning and extended 5-15cm from the catheter tip to engage each target chamber. Alternatively, the guidewire could be kept just inside of the catheter in order to stiffen it to advance the catheter into the chambers. The guidewire was oriented parallel to the z-axis in the cava and was interactively advanced and torqued to engage pulmonary artery branches to guide advancement of the catheter. Each chamber was sampled for hemoglobin oximetry.

As time (and subject patience) allowed, the procedure was repeated without a guidewire under conventional (normal-SAR bSSFP) imaging conditions. Subjects were observed for four hours after research catheterization, after which their research participation concluded.

Absent a safe method, guidewire tip heating was not directly measured in patients. Measures of systemic coagulation (plasma D-dimer immunoassay) and of focal red cell injury (spectrophotometric plasma free hemoglobin, Mayo Labs, Rochester, Minnesota, USA) were collected before and after CMR fluoroscopy catheterization, knowing they may be confounded by clinical conditions. Guidewire tips and catheter effluent were assessed for clot after every chamber sample, knowing that clot formation is common in guidewire catheterization without anticoagulation.

### Data analysis

Two independent investigators assigned Likert-type ordinal scales to measure catheter visualization (0 = invisible, 1 = sometimes visible, 2 = most times visible, 3 = all times visible) and confidence in catheter position (0 = uncertain, 1 = probable and requires pressure confirmation, 2 = certain) [5]. Guidewire visibility and confidence in tip position was also scored when the guidewire was extended from the catheter to engage each chamber.

Results are presented as mean ± standard deviation (SD) or median (1st quartile, 3rd quartile) as appropriate and are compared using a Wilcoxon rank sum test. A *p* value < 0.05 was considered statistically significant.

## Results

### Guidewire heating

Heating of metallic guidewires was influenced by CMR pulse sequence, guidewire configuration and guidewire physical properties, as summarized in Table 2.

**Table 2:** Factors that modulate guidewire heating during CMR

Factor	Heating Impact	How to reduce heating
CMR excitation energy		
Radiofrequency excitation, controlled via flip angle α	Heating increases with square of flip angle, α [14]	Reduce α during CMR fluoroscopy
Radiofrequency pulse width duration	Heating decreases linearly with RF pulse width duration	Increase radio frequenchy pulse width duration
Radiofrequency duty cycle	Heating decreases linearly with excitation repetition time (TR)	Prolong TR
Radiofrequency pre-pulses used for CMR magnetization preparation	Heating increases with pre-pulse flip angle and number of RF pulses in preparation, and decreases with RF pulse width duration	Reduce pre-pulses and their SAR characteristics
CMR scanner duty cycle	Additional heat is generated as long as CMR scanning continues	Limit duration of continuous CMR fluoroscopy with a guidewire in place.
Conductive guidewire physical properties		
Guidewire insulation	Insulation gaps, such as at the tip, concentrate current density and increase focal heating [8]	Use guidewires that are fully insulated without gaps
Length of conductive materials	Guidewire length > ¼ wavelength λ of the Larmor frequency in vivo (~ 10 cm at 1.5 T) promotes standing waves and therefore heating [13]	Use guidewires having metallic components shorter than ¼λ (not available commercially)
Guidewire configuration		
Guidewire position with regard to center of CMR bore	Electrical field minimal at the center of scanner bore (in x & y), greatest closer to wall of scanner bore [21]	Keep guidewire close to scanner centerline and away from walls of scanner.
Guidewire position with regard to patient body	Electrical field is greatest at outer (skin) surface of body. Electrical modeling suggests electrical field is greatest at groin and shoulder during CMR	Use in central blood vessels
Guidewire insertion length with regard to vascular access site	Electrical field is highest at outer edges of scanner bore entrance. Guidewire outside of the body is more likely to couple electrically and heat. In other words, minimal guidewire vascular insertion length is associated with maximal heating	Reduce input energy during scanning. Minimize time with guidewire at minimum insertion length.
Guidewire insulation by catheter	The patient is less exposed to guidewire heating when it is covered by insulating catheter	If guidewire is not in active use, retain its position inside catheter or remove from body during CMR.
Guidewire protrusion length from catheter	A change in insulation with minimum guidewire protrusion outside insulating catheters causes concentration of current density and therefore heating	Reduce input energy during scanning.
Guidewire length	Different guidewire lengths are associated with different degrees of heating, in a non-linear fashion, relating to coupling with scanner electrical field [21]	Select guidewire lengths empirically associated with less heating.
Guidewire diameter	Guidewires with smaller diameter generate more heating [8]	Select larger diameter guidewires as appropriate
Guidewire loops overlapping	Guidewire looping can create a second point of heating at wire contact points, which remains less than or equal to guidewire tip heating	Reduce input energy during scanning.
Guidewire heat is dissipated by conduction and convection into surrounding medium	Blood flow cools heated guidewire dramatically. Testing under static conditions, such as ASTM 2182 phantom, maximizes detected heating	Static phantom testing exaggerates heating to provide a margin of safety predicted in vivo, probably by 10-fold.

**Fig. 1:**
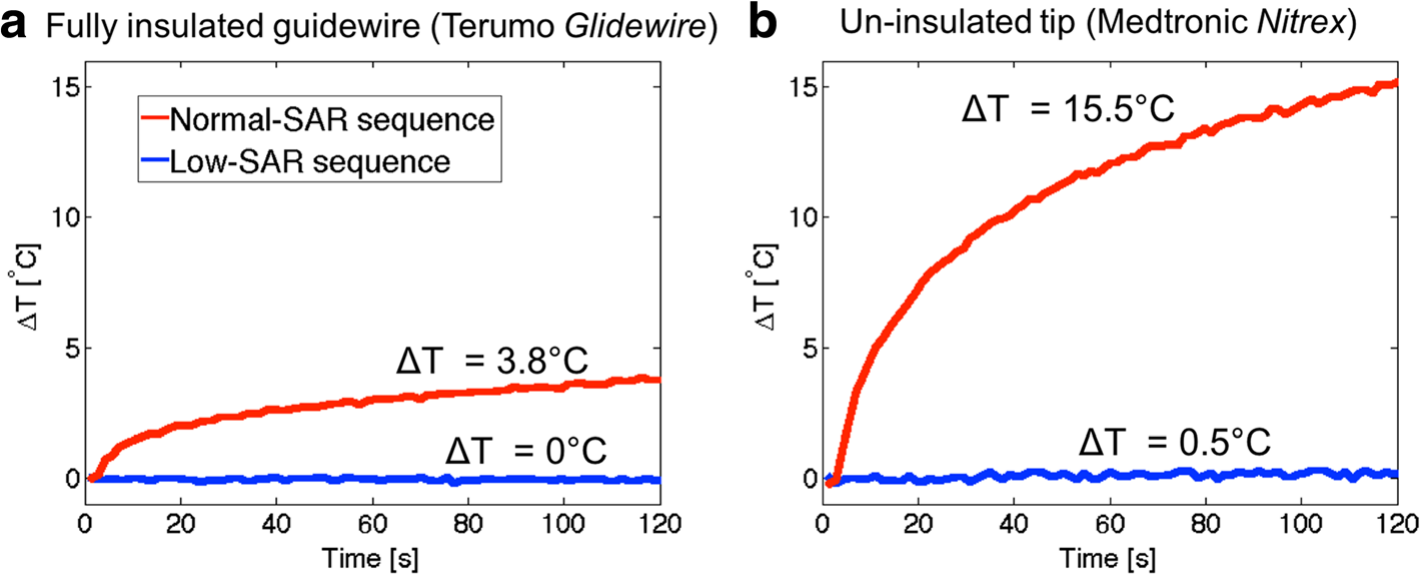
Heating of guidewires. Temperature at the tip of the fully insulated guidewire (Terumo *Glidewire*) (**a**) and uninsulated tip guidewire (Medtronic *Nitrex*) (**b**) during 2 min of continuous scanning (low-SAR and normal-SAR) in the ASTM 2182 phantom with homebuilt positioning system

The selected Terumo *Glidewire* was tested under worst-case heating conditions extended 1 cm from catheter tip positioned at the edge of the ASTM 2182 phantom (45 cm insertion, > 5 cm depth, 12.7 cm off-isocenter) in a straight configuration. It generated < 4°C with normal-SAR imaging and < 0.1°C with low-SAR imaging during 2 min of continuous imaging (Fig. 1a). Under identical conditions, the comparator *Nitrex* guidewire generated 15.5°C of heating with normal-SAR imaging and 0.5°C heating with low-SAR imaging during 2 min of continuous imaging (Fig. 1b). The incomplete insulation exposes metal at the tip of the nitinol guidewire concentrates current density and contributes to heating of this guidewire model [8].

Animal post-mortem experiments found maximum heating of 0.3°C with normal-SAR imaging and 0°C with low-SAR imaging during 2 min of continuous scanning. Maximum heating was observed as the guidewire protruded from the femoral sheath into the vasculature.

### Patients and clinical findings

Of 13 consecutive patients undergoing right heart catheterization during the study period, 8 consented and 7 underwent the study procedure. One was excluded because of hardware malfunction after vascular access was obtained; the operator felt the patient’s discomfort required swift conclusion of the procedure under X-ray. Two were excluded because they required emergency intervention, one declined CMR guidewire catheterization and underwent standard CMR fluoroscopy catheterization without a guidewire, and two required left but not right heart procedures.

Seven patients (46±20 years; 4 (57%) female) underwent guidewire CMR fluoroscopy catheterization. All had normal cardiac anatomy. All had suspected pulmonary hypertension. Underlying illness was sickle cell disease (*n* = 3); atrial septal defect (*n* = 2); cardiomyopathy (*n* = 1); and rheumatologic illness(n = 1). One had chronic renal failure and underwent catheterization using a gas-filled, instead of gadolinium contrast-filled, balloon catheter. One underwent nitric oxide vasodilator challenge under CMR. One underwent concomitant percutaneous coronary intervention and two underwent atrial sepal defect repair, all under X-ray guidance.

CMR fluoroscopy catheterization took a median of 30 (27, 48) minutes including catheter navigation, repeated procedure steps to measure chamber entry time and conspicuity using up to two imaging pulse sequences (low-SAR with guidewire and normal-SAR without guidewire), CMR flow, and in one case nitric oxide inhalation.

Hemodynamic and CMR findings are summarized in Table 3.

**Table 3:** CMR Catheterization Findings

Measurement	Value
Heart rate (bpm)	71 ± 10
Right atrial pressure (mm Hg)	9 ± 2
Right ventricular pressure (mm Hg)	42 ± 14 / 10 ± 5
Pulmonary artery mean / wedge pressure (mm Hg)	25 ± 9 / 12 ± 7
Aorta systolic/diastolic/mean pressure (mm Hg)	124 ± 19 / 67 ± 7 / 87 ± 9
Right atrium volume index (mL/m^2^)	14 ± 5
Right ventricular end-diastolic volume index / end-systolic (mL/m^2^) / ejection fraction	87 ± 37 / 35 ± 15 / 59 ± 5
Left atrial volume index (mL/m^2^)	40 ± 11
Left ventricular end-diastolic volume index / end-systolic (mL/m^2^) / ejection fraction	69 ± 6 / 26 ± 4 / 62 ± 6
Left ventricular mass index	54 ± 36
Q_P_ / Q_S_ / Ratio	3.6 ± 1.2 / 2.9 ± 0.9 / 1.3 ± .5

### Clinical performance of CMR fluoroscopy catheterization with- and without a guidewire

CMR guidewire fluoroscopy catheterization was successful in entering intended cardiac chambers in 100% of attempts, compared with non-guidewire fluoroscopy catheterization, 94% (p = NS). The failures were exclusively branch pulmonary arteries, both left and right.

Time-to-enter each chamber was not significantly different between the two approaches, low-SAR with guidewire and normal-SAR without guidewire (35 ± 45 s vs 26 ± 38 s, p = NS).

**Fig. 2:**
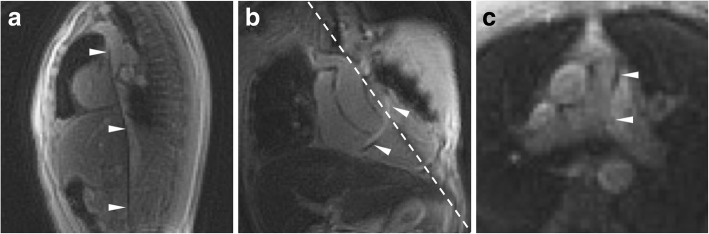
Catheter shaft conspicuity imparted by guidewire. Example low-SAR spiral GRE images, with catheter and guidewire (arrowheads) positioned in the superior vena cava (**a**), main pulmonary artery (**b**) and left pulmonary artery (**c**); the guidewire imparts both hypo- and hyper-intense signal along the shaft. The dotted line indicates signal saturation caused by an orthogonal slice plane interleaved during imaging

**Fig. 3:**
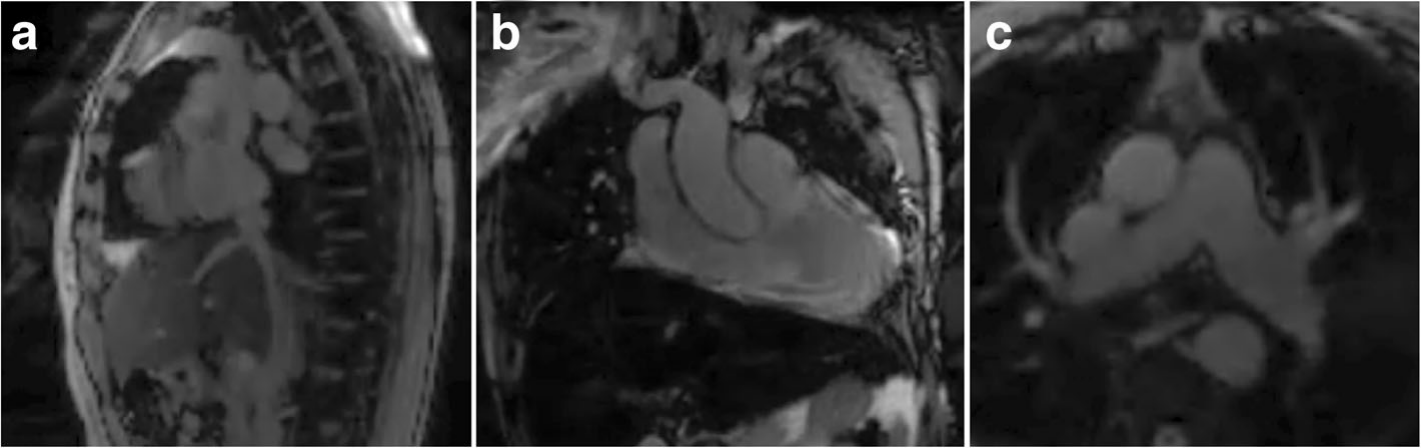
Comparator rectilinear bSSFP images. Image orientation comparable to Fig. 2 showing improved blood-tissue contrast with bSSFP rectilinear images (normal-SAR imaging mode) for inferior/superior vena cava view (**a**), main pulmonary artery view (**b**) and branched pulmonary arteries view (**c**). No devices were present during imaging


Table 4 shows Likert-type scores of catheter and guidewire tip and shaft visibility compared using the two different imaging techniques. Overall visibility and operator confidence in catheter tip location was comparable among all modalities using saturation pre-pulses. The catheter shaft was rendered slightly more visible by a guidewire susceptibility artifact (Fig. 2), but we observed that catheter shaft visibility was reduced when saturation pre-pulses were applied to confirm contrast-filled balloon position. Guidewire tip visibility was poor throughout. Imaging during navigation from inferior to superior vena cava is shown in Additional file 1: Video 1 available online. As expected, GRE imaging (low-SAR pulse sequence) provided inferior blood-myocardium contrast compared to bSSFP imaging (normal-SAR pulse sequence) (comparison images provided in Fig. 3).

**Table 4:** Likert-type scoring of device visibility and confidence in catheter and guidewire position. Only catheter shaft visibility reached statistical significance (*p* = 0.001)

Imaging test	Guidewire low-SAR spiral GRE	No guidewire normal-SAR rectilinear bSSFP
Tip visibility of balloon catheter with saturation pre-pulse(scale 0–3)	3.0 ± 0.2	2.9 ± 0.3
Shaft visibility of balloon catheter (scale 0–3)	1.1 ± 1.2	0.0 ± 0.0
Confidence that balloon catheter tip is at the intended location (scale 0–2)	2.0 ± 0.0	2.0 ± 0.0
Tip visibility of guidewire (scale 0–3)	0.5 ± 0.8	N/A
Shaft visibility of guidewire (scale 0–3)	1.6 ± 1.1	N/A
Confidence that guidewire tip is at the intended location (scale 0–2)	0.4 ± 0.6	N/A

**Additional file 1:** Video 1. (MP4 7886 kb)


### Safety

There were no adverse events in this small cohort.

To test for subclinical sequelae of guidewire heating, and out of caution, we measured baseline and follow-up markers of coagulation (plasma D-dimer) and of erythrocyte destruction (plasma free hemoglobin). These were all unchanged except for one patient who had undergone percutaneous coronary stenting using heparin anticoagulation, in whom plasma free hemoglobin rose from 6.4 to 15.2 mg/dL, which is the upper limit of normal.

We also inspected guidewires and catheter aspirates for thrombus formation after use. Clot was observed on the guidewire or catheter aspirate in 15% of guidewire specimens versus 11% of non-guidewire specimens (p = NS), and none among subjects who received heparin anticoagulation (one for percutaneous coronary stenting, two for left atrial catheterization).

## Discussion

Adoption of CMR fluoroscopy catheterization has been hampered by the unavailability of safe CMR-compatible clinical devices, especially guidewires. Metallic guidewires by definition are conductive and therefore prone to heating in the conditions required for CMR fluoroscopy catheterization in adults. Certain steps in right heart catheterization are difficult or impossible without a guidewire in some patients [2, 3, 5], especially selecting branch pulmonary arteries. The mechanical properties of commercial metallic guidewires are superior to non-metallic guidewires and we are not aware of any non-metallic guidewires with satisfactory mechanical properties for catheterization. To address concerns of metallic guidewire heating, we comprehensively tested and confirmed the safety of CMR catheterization using a specific guidewire under specific low-SAR imaging conditions, both in vitro and in animals before translating into human patients. Post-mortem animal experiments were chosen to exaggerate potential heating with no blood flow cooling. Heating of the selected guidewire with the low-SAR pulse sequence was negligible (< 0.07 °C) and less than the equipment accuracy of ±0.3 °C for all conditions tested in vitro.

This is the first in human report of CMR fluoroscopy cardiac catheterization with a standard commercially available metallic guidewire. The main findings of this study are that (1) a specific nitinol guidewire (*Glidewire* 0.035″ 150 cm angled-tip) can be used under specific low-SAR conditions to facilitate CMR fluoroscopy right heart catheterization at 1.5 T; (2) the guidewire imparts conspicuity to the shaft of otherwise-invisible polymer balloon-tipped catheters; (3) the incremental stiffness imparted to the polymer catheter facilitates procedure success under CMR fluoroscopy; (4) systemic measurements of coagulation (D-dimer) and erythrocyte injury (plasma free hemoglobin), although of unknown sensitivity, were not significantly perturbed using the guidewire; (5) clot accumulated on the guidewire and on guidewire-free catheters in patients undergoing CMR fluoroscopy when not anticoagulated, a known phenomenon under X-ray of unclear significance under these conditions; (6) Despite the reduced contrast-to-noise ratio, the low-SAR imaging mode was adequate to guide catheter navigation for CMR fluoroscopy right heart catheterization; and, (7) the visual performance of the specific nitinol guidewire used under CMR fluoroscopy is grossly inferior to X-ray in that the tip is not conspicuous, which is a common problem for passive devices in CMR [18] that must be considered carefully by operators.

Ours is not the first human interventional CMR procedure using a Terumo guidewire device. Paetzel et al. reported femoropopliteal balloon angioplasty in 15 patients under real-time CMR guidance at 1.5 T in 2005 [19]. They used low-SAR gradient echo pulse sequences and assured adequate insertion length and proximity to isocenter before CMR, and found no heating related events. The authors did not report on guidewire visibility, which presumably was as poor as in our experience.

### Recommended workflow

Our approach would be simple to replicate at other medical centers. We describe a procedure for nitinol guidewire-assisted CMR fluoroscopy right heart catheterization using specific conditions at 1.5 T. We believe this approach should be only undertaken by expert catheter operators familiar with this operating environment to avoid (1) inadvertent guidewire scanning using normal-SAR conditions (high flip angle, short TR), (2) tissue injury or myocardial perforation caused by inadequate guidewire tip visualization despite tactile cues, (3) selection of an alternative commercial guidewire with unsafe heating properties.

Based on this experience, we intend to employ guidewires (with low-SAR CMR fluoroscopy) catheterization only when needed, much as they are employed during X-ray fluoroscopy catheterization. We recommend other sites adopt a workflow that clearly assures low-SAR imaging modes during guidewire steps, such as explicit team communications, checklists, and visual warnings on imaging displays. We also recommend routine low-dose anticoagulation which is widely employed by pediatric but not by most adult catheterization programs, in light of the low but appreciable incidence of clots recognized in all arms of this study.

We performed this work in a combined X-Ray/CMR suite, although this procedure would also be feasible in a standard 1.5 T CMR suite with an appropriate evacuation plan in case of emergency. A CMR compatible defibrillator [20] might be helpful for complex CMR-guided procedures.

### Limitations

We have not yet tested CMR fluoroscopy guidewire catheterization during retrograde left heart catheterization in patients, and we caution against such procedure absent assurance about guidewire tip visualization during CMR fluoroscopy. We also warn against the likely danger of using guidewires other than the specific device described unless carefully examined for heating. The specific device has insulation and length properties and we were able to demonstrate freedom from heating under the conditions of use.

Visualization of commercial nitinol guidewires remains a challenging, though work in positive contrast imaging methods may improve the visibility at the expense of temporal resolution [14]. We chose an 8 mm slice thickness to avoid out-of-plane guidewire motion, but thinner slices could be explored to improve visualization of the signal void.

## Conclusion

Metallic guidewires are an important adjunct for catheterization as they interactively control shaft stiffness, and help to select and enter target chambers. By lowering SAR (through reduced RF excitation flip angle and increased repetition time, implemented as spiral gradient echo CMR), and by selecting a specific fully-insulated nitinol guidewire, we safely employed a metallic guidewire during CMR fluoroscopy catheterization. The guidewire also improved shaft conspicuity, which we believe facilitated procedure success. These findings should not be applied to other nitinol guidewire devices or similar devices having different lengths without specific testing to assure freedom from heating. Though the guidewire visual performance is inferior to X-ray in that the tip is not conspicuous, the ability to safely use a metallic guidewire during CMR fluoroscopy could enable new and more complex CMR-guided procedures.

## Abbreviations

bSSFP: Balanced steady state free precession
CMR: Cardiovascular magnetic resonance
FOV: Field-of-view
GRE: Gradient recalled echo
LV: Left ventricle
NS: Non-significant
PTFE: Polytetrafluoroethylene
RF: Radiofrequency
RHC: Right heart catheterization
RV: Right ventricle
SAR: Specific absorption rate
TR: Repetition time
α: Flip angle
λ: Wavelength
